# Evolution of the Structural, Mechanical, and Phonon Properties of GeSe Polymorphs in a Pressure-Induced Second-Order Phase Transition

**DOI:** 10.3390/ma12213612

**Published:** 2019-11-03

**Authors:** Jianhui Yang, Qiang Fan, Bing Xiao, Yingchun Ding

**Affiliations:** 1 School of Physics and Electronic Engineering, Leshan Normal University, Leshan 614004, China; yjh20021220@foxmail.com; 2 State Key Laboratory of Electrical Insulation and Power Equipment & School of Electrical Engineering, Xi’an Jiaotong University, Xi’an 710049, China; bingxiao84@xjtu.edu.cn; 3 College of Optoelectronic Technology, Chengdu University of Information Technology, Chengdu 610225, China; dyccqzx@cuit.edu.cn

**Keywords:** phase transition, first principle calculations, elastic constant, phonon mode softening

## Abstract

A pressure-induced structural transition from the layered-like phase (*Pnma*) to another bilayer structure (*Cmcm*) in GeSe was investigated with first principle calculations. The variations of the structural, electronic, elastic, and vibrational properties of GeSe with the application of pressure were obtained. The transformation from the *Pnma* to *Cmcm* phase occurred at 34 GPa. The *Cmcm* phase structure showed dynamical stability above 37 GPa. The lattice parameters and the equation of state varied continuously at the transition pressure. Obvious stiffening in the *C*_33_ and *C*_23_ elastic constants associated with the compressive and shear components was observed to occur within the phase transition process. Two characteristic Raman modes (A_g_ and B_3g_) of the *Pnma* phase showed significant softening by increasing the pressure.

## 1. Introduction

**Figure 1:**
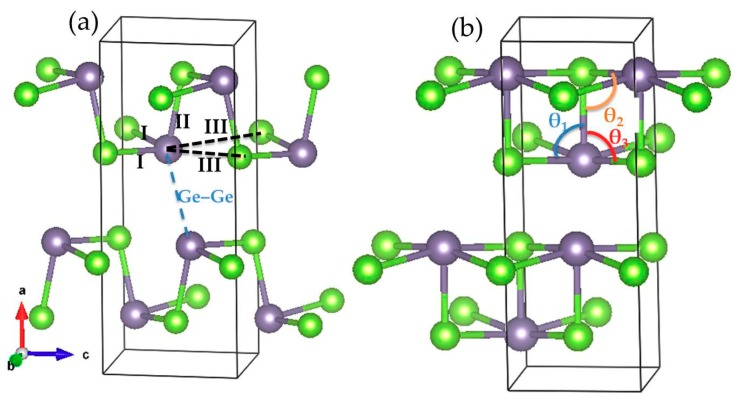
Crystal structure of GeSe with the (**a**) *Pnma* and (**b**) *Bbmm* of GeSe. The nearest Ge–Se bonds are shown with double color lines.

GeSe, a typical bilayer IV-VI compound, has attracted much attention with respect to thermoelectric [1,2], optical [3], optoelectronic [4], and photovoltaic applications [5] because of its low thermal conductivity, excellent electrical and optical properties, as well as excellent thermal stability and low-toxic constituent elements. At atmospheric pressure, GeSe crystallizes into an orthorhombic structure with a *Pnma* space group. The unit cell contains eight atoms, arranged in two layers, and each layer consists of four atoms; all of them are located at the 4c Wyckoff site (the crystal structure is shown in Figure 1a. The atoms within the layer form strong covalent Ge–Se bonds, and each Ge atom is coordinated with the other three nearest Se atoms. Additionally, the interlayer is piled up with weak van der Waals interaction along the a axis, and it forms a zigzag Ge–Se chain along the c axis.

Recently, more effort has been put into understanding the structural phase transition mechanism of GeSe under high pressure [6,7,8]. In one of the earliest studies, Bhatia et al. [9] claimed that they observed a phase transition of GeSe from a non-metallic orthorhombic structure to a metallic rock salt structure at 6 GPa. Hsueh et al. [10] reported that they could not locate the structural phase transition up to 13 GPa, but the predicted electronic structure transformation from a non-metallic into metallic state was about 6 GPa. In addition, Onodera et al. [11] also argued that GeSe could retain orthorhombic symmetry up to 82 GPa, but it showed metallic behavior above 25 GPa. Recently, Xu et al. used XRD to study the structural transition of GeSe at high pressure. They observed a continuous phase transition from orthorhombic (*Pnma*) to orthorhombic (*Cmcm*) occurring in a wide pressure range (25–35 GPa), and the structural phase transition completed near 40 GPa [7].

Previous theoretical and experimental studies on the high-pressure behaviors of GeSe polymorphs have led to controversies regarding the detailed phase transition mechanism. The variations of the structural parameters, elastic constants, and phonon properties during the phase transition also lack investigation in the literature. In our work, we performed systematic first principle calculations on the *Pnma* to *Cmcm* transformation of GeSe polymorphs within the application of hydrostatic pressure from 0 to 50 GPa to reveal the origin of the phase transition mechanism.

## 2. Computational Methods and Details

All the first principle calculations were performed in the framework of density functional theory (DFT) with the projector-augmented wave method under the periodic boundary conditions described in VASP [12]. The exchange–correction functional was described with a generalized gradient approximation (GGA). Considering the van der Waals interaction between the two layers, we adopted an optB88-vdW functional to correctly describe the van der Waals interactions [13]. The kinetic energy cutoff for the plane-wave basis was set to 600 eV. The Γ-point centered Monkhorst–Pack k-mesh of 5 × 15 × 15, which included 192 irreducible k-points in the first irreducible Brillouin zone, was used for the structural relaxation and energy calculations. The convergence criteria for the atomic force and the total energy were 10^−3^ eV/Å and 10^−6^ eV, respectively. The primitive structure was fully relaxed at zero pressure to gain the optimized structure. With the same optimization approach, the hydrostatic pressure was applied to the optimized structure at zero pressure and obtained the fully relaxed structure at the certain pressure. To outline the evolution of structural features in the pressure-induced structural phase transition, the hydrostatic pressure was gradually applied to 50 GPa. Using the standard strain–stress method [14], the elastic constants of GeSe were obtained in the same computational program, VASP.

The phonon spectra were computed using the finite-displacement method as implemented in the PHONOPY code [15]. The atomic forces were calculated using a 2 × 2 × 3 supercell and a 1 × 2 × 2 k-point grid. The other computational parameters were the same as those of the aforementioned bulk calculations.

## 3. Results and Discussion

### 3.1. Structural Phase Transition

Starting from the reports, the geometry optimization of GeSe in the *Pnma* phase under zero hydrostatic pressure was carried out. The equilibrium structural parameters, including the lattice constants and the atomic coordinates, were obtained without the pressure effect. The optimized crystallographic data of *Pnma* GeSe under zero pressure are listed in Table 1.

The optimized structure data under zero pressure are in good agreement with the values published in the previous theoretical results [17,18]. Taking into account the overestimation of the lattice constants by the GGA approximation, the results agree fairly well with the experimental values [1,16]. In the *Pnma* phase, the Ge and Se atom sit in the Wyckoff position 4*c* (x, 1/4, z), which is fixed y = 1/4. The fractional coordinates of the Ge and Se atom are well reproduced in the present work. The Ge atom bonds with the three nearest neighbor Se atoms. The bond of the Ge–Se in the layer plane is denoted as I, and the bond nearly perpendicular to the layer plane is represented as II. In addition to the three nearest Ge–Se bonds, the Ge has two next-nearest nonbonding Se neighbors in the layer plane, indicated by III as illustrated in Figure 1a. The blue dashed line represents the nonbonding interlayer Ge–Ge distance. The calculated three nearest bond lengths of Ge–Se are I = 2.61 Å and II = 2.59 Å. The next-nearest bond length of Ge–Se III is 3.38 Å. The calculated bonding and nonbonding lengths display satisfactory agreement with the previous experimental and theoretical reports [1,7]. Based on the equilibrium structure under zero pressure, we gradually increased the hydrostatic pressure to observe the structural evolution behavior under pressure. The structural parameters of GeSe were optimized completely under different pressures. We found that the structure symmetry of GeSe changes at 34 GPa from a low-symmetry space group *Pnma* (#62) to high-symmetry *Bbmm* (#63). The *Bbmm* space group has the same crystal axis as the *Pnma* phase group, which is a non-standard *Cmcm* (#63) space group. Both *Pnma* and *Bbmm* occupy the 4c Wyckoff positions at (x, 1/4, z). The 4c Wyckoff positions of group *Pnma* are (x,1/4,z), (−x + 1/2,3/4,z + 1/2), (−x,3/4,−z), and (x + 1/2,1/4,−z + 1/2). The Wyckoff positions of group *Bbmm* are (x,1/4,0), (−x + 1/2,3/4,1/2), (−x,3/4,0), and (x + 1/2,1/4,1/2). The *Cmcm* phase structure is dynamically stable at 37 GPa, as discussed below. The finding reveals that the second-order phase transition of GeSe is induced by pressure. The *Bbmm* phase at high pressure is illustrated in Figure 1b, which also shows a layered structure with eight atoms in a unit cell. In the *Bbmm* phase, the coordination number of each Ge atom is five, compared to three of the low-pressure *Pnma* phase structures. The lattice parameters of the *Bbmm* phase at 37 GPa are a = 9.74 Å, b = 3.57 Å, and c = 3.68 Å. The four neighbor bond lengths of the Ge–Se in the layer plane are I = III = 2.57 Å. The bond length of the Ge–Se–II perpendicular to the layer plane is 2.44 Å. On the experimental site, Xu et al. [7] observed a high-symmetry *Bbmm* phase under 40 GPa, with a = 9.65 Å, b = 3.58 Å, c = 3.61 Å, I = III = 2.56 Å, and II = 2.44 Å. 

**Figure 2:**
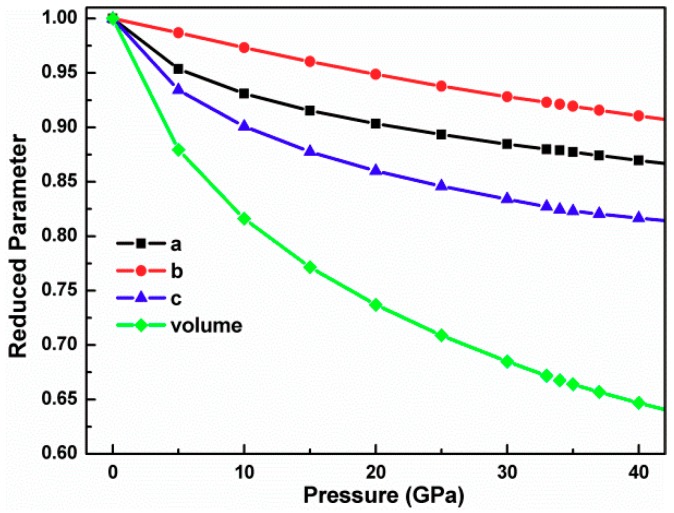
Dependence of the reduced crystal parameters of GeSe on the hydrostatic pressure.

The pressure dependence of the lattice parameters, cell volume, bond length, and bond angle may provide useful information regarding the original phase transition. To investigate further details of the effects of pressure, the reduced crystal parameters including lattice constants and cell volume as a function of hydrostatic pressure are plotted, as shown in Figure 2.

**Figure 3:**
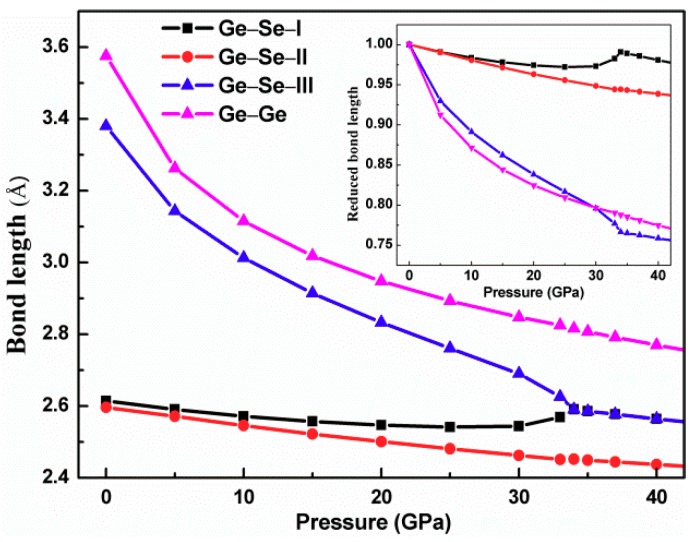
Variations of the bond lengths as a function of the hydrostatic pressure.

During the phase change process with the application of pressure, the orthorhombic structure is maintained. The lattice parameter decreases monotonically with the application of the pressure and the compression has strong anisotropy. The reduced lattice parameters a, b, and c at 35 GPa are 0.877, 0.919, and 0.823, respectively, which implies that the main compression is along the c axis, namely, in the zigzag puckered direction. Similar behavior was discovered in layered crystal [19]. It is worth mentioning that the cell volume shows a continuous monotone decrease with the increasing pressure with no abrupt changes. The variation of the cell volume with the pressure suggests that the atomic rearrangement is intensive. Thus, the transformation process is managed gradually. The dependence of the pressure on the neighbor Ge–Se and Ge–Ge bond lengths, marked in Figure 1a, is depicted in Figure 3.

Accordingly, as seen in Figure 3, the length of the Ge–Se–II bond nearly perpendicular to the layer plane decreases linearly with pressure. The Ge–Se–III nonbonding distance in the layer plane decreases rapidly with the increase of the pressure and it reaches the same value as the Ge–Se–I bond (2.59 Å) at 34 GPa. When the pressure increases up to 34 GPa, the decrease rate of bond III is essentially linear with the equal value of bond II. The distance of the Ge–Ge nearly perpendicular to the layer plane decreases sharply with the increase of the pressure, similarly to the Ge–Se–III bond. Consequently, the symmetry of the GeSe structure becomes higher when the hydrostatic pressure approaches 34 GPa. To show the effect of the hydrostatic pressure on the bond distance more clearly, the relationship between the reduced bond distance and the pressure is plotted in the inset of Figure 3. It is obvious that the nonbonding distances of the intralayer Ge–Se and the interlayer Ge–Ge are shortened more quickly than the other bond lengths with the increasing pressure. When the pressure reaches 34 GPa, both the bond length of Ge–Se–III and the distance of Ge–Ge decrease approximately linearly with the increase of pressure. Moreover, we further analyzed the evolution of the bond angles θ_1_ (Se–Ge–Se), θ_2_ (Ge–Se–Ge), and θ_3_ (Se–Ge–Se) as marked in Figure 1b with the pressure application. The bond angles θ_1_ and θ_2_ decrease slowly and monotonically with the pressure. In contrast, the bond angle θ_3_ become larger gradually with the increase of pressure. The bond angles θ_1_, θ_2_, and θ_3_ at zero pressure are 92.41°, 102.63°, and 76.76°, respectively. When the pressure reaches 37 GPa, the bond angles of θ_1_ and θ_2_ decrease to 83.69° and 96.30°, whereas the angle of θ_3_ increases up to 83.69°. These findings suggest that the zigzag Ge–Se chain along the c axis gradually disappears with the increasing pressure. The mechanism of compression of GeSe under pressure is mainly due to the narrowing of the Ge–Se distance in the layer plane and the interlayer Ge–Ge separation. The evolution of the 4c Wyckoff positions at (x, 1/4, z) of the Ge and Se atom are illustrated in Table 2.

Table 2 clearly shows the evolution of the 4c Wyckoff positions at (x, 1/4, z) with the variation of the pressure. The 4c Wyckoff positions x and z of Ge and Se are pressure dependent. The fractional coordinate of Ge(z) monotonically decreases with the increasing pressure, whereas the Se(z) increases robustly. When the pressure reaches 34 GPa, Ge(z) and Se(z) approach the high-symmetry values of 0.0 and 0.5, rendering Ge–Se–I and Ge–Se–III equal in the *Cmcm* phase. In addition, the pressure dependence of the fractional coordinates from 0 GPa to 5 GPa is sharper than the others, which is similar to the pressure dependences of the lattice parameters and bond lengths. The evolution of bond length and fractional coordinates outlines a displacive space group transformation from *Pnma* (62) to *Cmcm* (63) for GeSe with applied high pressure.

### 3.2. Electronic Properties

**Figure 4:**
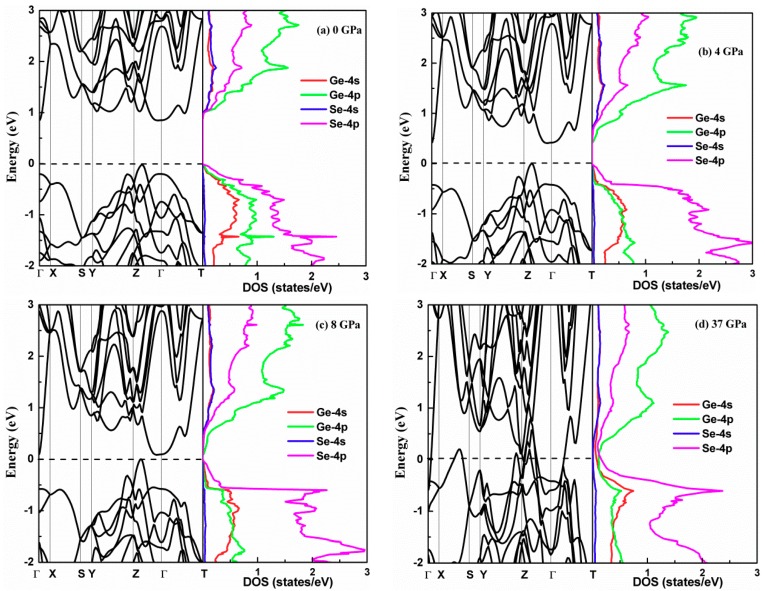
Band structure and projected density of states (DOS) of GeSe calculated at increasing simulated pressures: (**a**) 0 GPa; (**b**) 4 GPa; (**c**) 8 GPa; (**d**) 37 GPa.

Next, we explored the electronic structure of GeSe under pressure. Figure 4 plots the electronic band structure of GeSe under different simulated pressures, and the projections of the s and p orbitals for the Ge and Se atoms are shown as well. The energy band structure is plotted along the high-symmetry line Γ-X-S-Y-Z-Γ-T. The zero-band energy line is set to the top of the valence band. We observed that the first valence band maximum (VBM) is located on the Γ-Z line and the second VBM and the conduction band minimum (CBM) site on the Γ point. The energy difference between the two maximum valence bands is 0.14 eV. The results confirm that GeSe in the *Pnma* phase is an indirect bandgap semiconductor. The computed indirect and direct bandgaps of GeSe at 0 GPa are 0.89 eV and 1.03 eV, which is slightly smaller than the data measured from the experiment at approximately 1.14 eV and 1.21 eV [20,21]. However, the results are in excellent agreement with the previous predicted values of 0.85 eV and 0.98 eV [17] based on the first principle calculation. It is well known that the band gap is always underestimated in first principle calculations, and the main reason is that the derivative discontinuity of the exchange correlation energy between integer electrons is not captured in DFT. The projected density of states (DOS) shows that the contributions on conduction states mainly originate from Ge-4p and Se-4p. The Ge-4s and Se-4s contribute equally to conduction states, but the contribution from Se-4s nearly equal to zero. The valence states are mostly originated from Se-4p orbitals. The band profile under pressure is similar to the band structure at 0 GPa. The energy bandgap of GeSe decreases rapidly under pressure. The energy bandgap under 8 GPa is only 0.1 eV.

**Figure 5:**
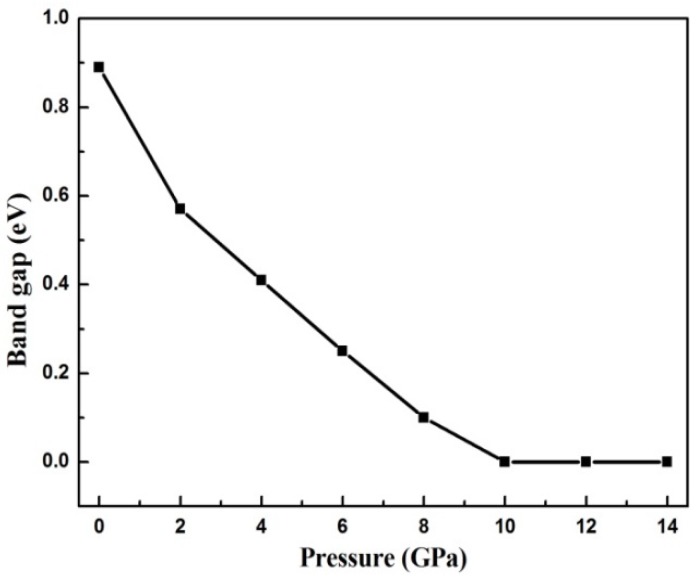
Relationship between the energy bandgap of GeSe and the applied pressure.

According to Figure 5, the applied pressure narrows the bandgap significantly, and the bandgap closes under 10 GPa. The bandgap remains at zero as the pressure increases above 10 GPa, comparable with previous research [22]. This indicates that the pressure induces the transition from a semiconductor to a metal in GeSe. It is noteworthy that the metallization occurring at 10 GPa does not give rise to symmetry changes. A similar phenomenon appears in the homologous compounds [23,24,25,26]. It should be noted that, as shown in Figure 5, the bandgap below 2 GPa falls more dramatically than that above 2 GPa, which is in accordance with the relationship between the lattice constant and the pressure.

**Figure 6:**
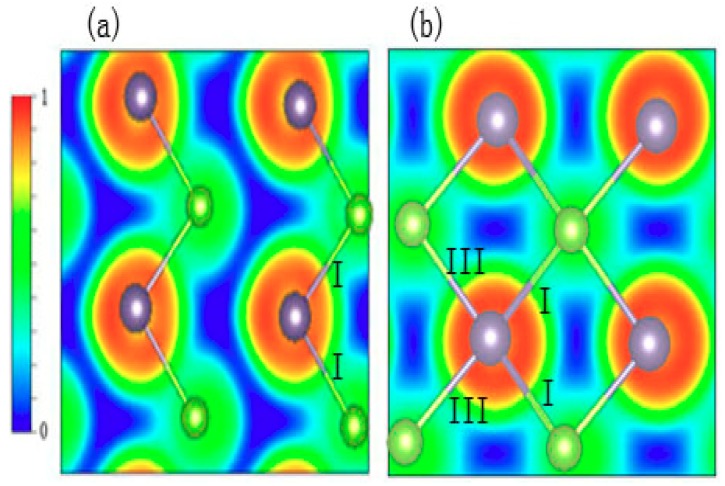
The ELF values of GeSe projected to a layered plane under (**a**) 0 GPa and (**b**) 37 GPa.

The structural features show that when the transition takes place, the coordination number of each Ge atom changes from three to five. In order to reveal the bonding mode during the phase transition, the bonding profile is directly illustrated through the electron localization function (ELF). The localizations of electrons for different bonding types are noticeably different from each other. For example, the electrons are located between two atoms in a covalent bond, and they are distributed only near the anion in an ionic bond, whereas the electrons are completely delocalized in metal. The electron localization characteristics are revealed with the ELF value. The ELF’s possible range of value is from 0 to 1. The electrons are perfectly localized when the ELF value approaches 1, and they become more delocalized with a lower value [27,28]. To gain further insight into the nature of the behavior of the chemical bond characteristics in the phase transition process, the ELF of GeSe at different pressures was investigated in real space. The ELF profiles projected to a layered plane under 0 GPa and 37 GPa are shown in Figure 6.

At zero pressure, the density of the localized electrons along the Ge–Se–I bond is steady, and the value of the ELF along bond I is approximately 0.4, which suggests that bond I forms a covalent bond. The blue region along the long Ge–Se–III bond indicates low density of the localized electrons, which demonstrates that the covalent bond fails to form because there are almost no valence electrons in the long Ge–Se pairs. The hydrostatic pressure slightly changes bond I and it significantly compresses bond III as noted above, driving the phase transition. As can be seen in Figure 6b, at high pressure, there are four equivalent Ge–Se pairs in a layer. The atomic structure model illustrates the fact that the four bonds have the same bond length. The ELF profile also shows the density of the localized electrons, which are uniformly distributed between Ge–Se bonds. Furthermore, compared with the profile of the ELF between the paired Ge–Se atoms in bond I under different pressures, the hydrostatic pressure slightly reduces the density of the localized electrons between the paired Ge–Se atoms.

### 3.3. Elastic and Mechanical Properties

Elastic constants provide more detailed information during the phase transition process. The elastic constants were calculated using the symmetry-dependent strain–stress method. The calculated elastic constants for GeSe under an external hydrostatic pressure up to 50 GPa are illustrated in Table 3.

It was found that all the elastic constants increased with the increasing external hydrostatic pressure. This pressure dependence characteristic of the elastic constants could be attributed to the covalent bond behavior with the increasing pressure. The *C*_11_, *C*_12_, and *C*_33_ from 0 GPa to 50 GPa increase approximately ten times. The *C*_22_, with the slowest growth rate, also increases by a factor of 3.5 with the applied external pressure. Moreover, the values of *C*_33_ and *C*_23_ have an obvious jump near 34 GPa. These elastic constant jumps at phase transition pressure are typical in second-order continuous structure phase transitions [29,30]. The elastic constants *C*_11_, *C*_22_, and *C*_33_ represent the uniaxial compressive elasticity along the [100] (a axis), [010] (b axis), and [001] (c axis) directions, respectively. The elastic constant *C*_22_ is larger than *C*_11_ at 0 GPa. With the increase of the external pressure, the elastic constant *C*_11_ increases rapidly and exceeds *C*_22_ at 10 GPa. The elastic constant *C*_22_ increases monotonously with the applied pressure and it is insensitive to the phase transition. It can be seen that *C*_33_ > *C*_22_ in the *Pnma* phase (below 33 GPa), whereas *C*_33_ is close to *C*_22_ in the *Cmcm* phase (above 34 GPa), implying that there is less elasticity along the c axis in the *Pnma* phase. The weaker elasticity indicates weaker atomic bonds. Therefore, deformation occurs more easily along the c axis, in accordance with the previous discussion about structural phase transitions. However, the elastic constants *C*_12_ (*C*_13_, *C*_23_) and *C*_44_ (*C*_55_, *C*_66_) represent shear elasticity in two dimensions. For example, *C*_44_, *C*_55_, and *C*_66_ are relative to the shear elasticity in the (100), (010), and (001) planes. In the studied pressure range, *C*_55_ > *C*_66_ > *C*_44_, implying that the shear transformation in the (010) plane is more difficult than that in other planes.

It is necessary to analyze the mechanical stability of the second-order phase transition. The mechanical stability criteria of an orthorhombic crystal with restricted elastic constants and bulk modulus are expressed as follows [31]:

(1) $$C_{11}>0\;,\;\;C_{11}C_{22}>C_{12}^{2},$$

(2) $$(C_{11}C_{22}C_{33}+2C_{12}C_{13}C_{23}-C_{11}C_{23}^{2}-C_{22}C_{13}^{2}-C_{33}C_{12}^{2})>0,$$

(3) $$C_{44}>0\;,\;\;C_{55}>0\;,\;C_{66}>0\;.$$

It is obvious that the elastic constants of GeSe under the applied external hydrostatic pressure fulfill the above criteria, suggesting that both the *Pnma* and *Cmcm* phases are mechanically stable.

### 3.4. Dynamical Stability and Optical Active Phonon Modes

**Figure 7:**
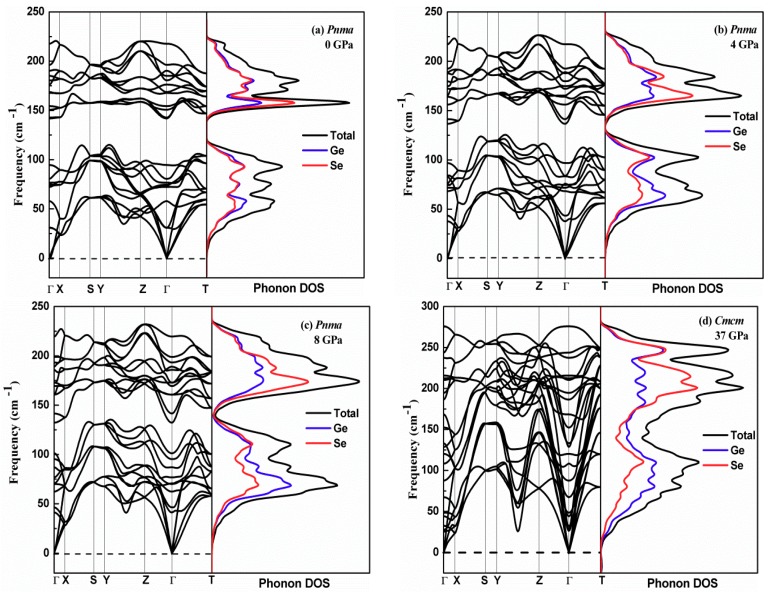
Phonon dispersion and phonon density of states (DOS) of GeSe along the high-symmetry directions at different pressures: (**a**), (**b**) and (**c**) at 0 GPa, 4 GPa and 8 GPa in *Pnma* phase, respectively; (**d**) at 37 GPa in *Cmcm* phase.

To assess the behavior of the dynamical stability of GeSe under pressure, the full phonon spectrum at different applied pressures up to 50 GPa was calculated using first principle calculations. The phonon dispersion curves along the high-symmetry path and the phonon density of states (PDOS) of GeSe at atmospheric pressure are presented in Figure 7a. No imaginary frequency is found, implying that the *Pnma* structure of GeSe at atmospheric pressure is dynamically stable. As mentioned above, the *Pnma* symmetry changes to higher *Cmcm* symmetry when the pressure reaches 34 GPa. However, the imaginary frequency of the acoustic branch appears in the phonon dispersion relationships at 34 GPa. Moreover, the imaginary frequency is located at the Γ point, not at other high-symmetry points in the Brillouin zone. The magnitude of the imaginary frequency decreases as soon as the pressure increases. The imaginary frequency disappears entirely when the simulated external pressure reaches 37 GPa, as seen in Figure 7d.

It is well known that the characteristics of phonon spectra are determined by both the chemical bond strength and the mass of the unit structure. It can be clearly seen in Figure 7a that there is an obvious gap in the phonon spectra of GeSe in the *Pnma* structure, from 118 cm^−1^ to 142 cm^−1^. The gap gradually decreases with the application of hydrostatic pressure. The minimum point of high-frequency optical phonons exists at Γ, which decreases with pressure. Additionally, the phonon spectra of GeSe in the *Cmcm* structure are practically merged. The frequencies of the acoustic vibrational modes along the Γ-Z direction increase, mixing with optical phonons, which is due to the enhancement of interlayer interaction. The vibrational branches of the *Cmcm* structure have significant dispersion, whereas the branches of the *Pnma* structure are almost flat. It is interesting to note that the highest phonon spectrum frequency (275.9 cm^−1^) of the *Cmcm* structure is higher than that of the *Pnma* structure (220.6 cm^−1^). The increase of the Ge–Se bond strength and the shortening of the bond length are responsible for this phenomenon.

**Figure 8:**
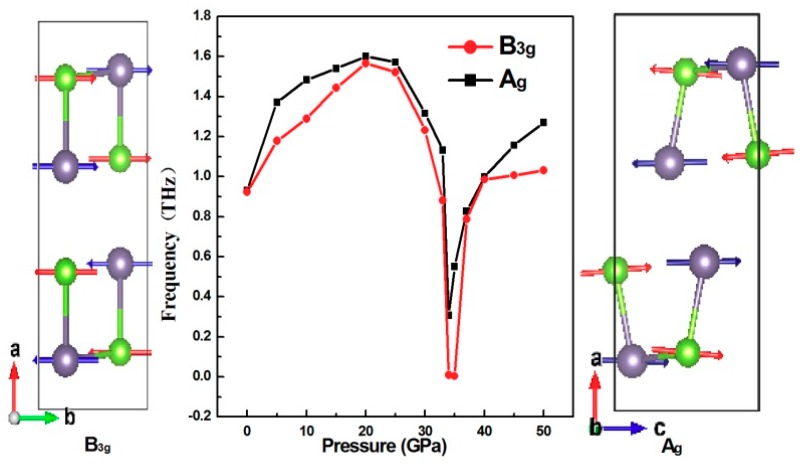
Hydrostatic pressure dependence of the calculated Raman-active B_3g_ and A_g_ shear phonon modes. The corresponding phonon eigenvectors are shown together with the VESTA code.

To assess the behavior of all phonon modes at the Brillouin zone center, we analyzed the pressure dependency of the lattice vibrations at the Γ point. According to group theory, the vibrations at the Γ point in the *Pnma* phase have the following phonon modes:

(4) $$\Gamma_{tot}\left(Pnma\right)=4A_{g}\left(R\right)+2A_{u}+2B_{1g}\left(R\right)+4B_{1u}\left(IR\right)+4B_{2g}\left(R\right)+2B_{2u}\left(IR\right)+2B_{3g}\left(R\right)+4B_{3u}\left(IR\right),$$

where R and IR correspond to the Raman and IR-active modes, respectively. $A_{u\;}$ is neither the Raman nor IR-active mode; it is called the silent mode. The zero frequency acoustic vibration modes correspond to the B_1u_, B_2u_, and B_3u_ modes, and the rest are optical modes. Usually, the phonon frequency increases with the pressure increase because the force constant between atoms increases with the decrease of volume. However, the calculated results of the pressure dependency of the lattice vibrations at the Γ point of GeSe show that the two lowest frequency Raman-active phonon modes A_g_ and B_3g_ experience remarkable change at 34 GPa. The pressure dependency of the phonon frequencies of the Raman-active modes A_g_ and B_3g_ and the relevant calculated phonon eigenvectors are illustrated in Figure 8. It is well known that the phonon vibration mode is caused by atomic vibrations. In order to obtain essential information about the atomic displacements giving rise to the vibration modes, the relevant phonon eigenvectors for A_g_ and B_3g_ are shown together in Figure 8.

The displacement pattern associated with the B_3g_ phonon mode corresponds to a shearing motion along the b direction. The A_g_ phonon mode is associated with displacement in the crystallographic c axis. It can be clearly seen that A_g_ and B_3g_ exhibit softening near the phase transition pressure at 34 GPa. This pressure-induced softening of the low-frequency symmetric mode can be found in other second-order phase transitions [32,33]. From the results, it is evident that a second-order phase transition takes place, with the transition of crystal symmetry from a simple orthorhombic phase (*Pnma*) to a high, centered orthorhombic phase (*Cmcm*). The phase transition is a displacement type induced by the softening of the low-frequency interlayer phonon mode.

## 4. Conclusions

In this work, we investigated extensively the pressure-induced structural, mechanical, and vibrational properties of GeSe using first principles based on the DFT. The pressure dependencies of the structural parameters including the lattice parameters, bond lengths, and atomic coordinates reveal the characteristics of the continuous second-order phase transition of GeSe from *Pnma* to *Cmcm* at 34 GPa. The electronic properties undergo significant change under applied pressure. The band gap of GeSe becomes narrow, and it ultimately closes at 10 GPa. The structural stability of GeSe in the *Pnma* and *Cmcm* phases is confirmed by the elastic constants and the phonon spectra. The pressure dependency of the elastic constants and the vibration phonon at the center point reveal the origin of the second-order phase transition mechanism in GeSe. The group theory analysis shows that the phase transition is induced by the softening of the low-frequency phonon mode in the interlayer.

## Figures and Tables

**Table 1:** Crystallographic data for *Pnma* GeSe under zero pressure.

Parameter	This Work	Experiment (Ref. [1])	Experiment (Ref. [16])	Calculation
a (Å)	11.15	10.83	10.83	11.17 ^a^, 11.01 ^b^
b (Å)	3.89	3.83	3.83	3.88 ^a^, 3.85 ^b^
c (Å)	4.47	4.39	4.39	4.52 ^a^, 4.47 ^b^
Ge (4*c*)	(0.1253, 1/4, 0.1208)	(0.1229, 1/4, 0.1106)	(0.1115, 1/4, 0.1211)	(0.127, 1/4, 0.098) ^b^
Se (4*c*)	(0.8536, 1/4, 0.4936)	(0.8549, 1/4, 0.5013)	(0.8534, 1/4, 0.5020)	(0.855, 1/4, 0.517) ^b^

The values denoted with superscript a and b come from Ref. [17] and [18], respectively.

**Table 2:** 4c Wyckoff positions at (x, 1/4, z) of Ge and Se under pressure.

Pressure	Ge(x)	Ge(z)	Se(x)	Se(z)
0	0.1253	0.1208	0.8536	0.4936
5	0.1175	0.0998	0.8566	0.4906
10	0.1142	0.0860	0.8577	0.4897
15	0.1125	0.0736	0.8583	0.4897
20	0.1115	0.0618	0.8589	0.4901
25	0.1110	0.0492	0.8595	0.4913
30	0.1107	0.0341	0.8601	0.4933
33	0.1109	0.0136	0.8607	0.4972
34	0.1109	0.0000	0.8608	0.5000
35	0.1106	0.0000	0.8608	0.5000
37	0.1103	0.0000	0.8608	0.5000
40	0.1098	0.0000	0.8609	0.5000

**Table 3:** Calculated elastic constants (*C*_ij_ in GPa) of GeSe under the applied external hydrostatic pressure.

Pressure	*C*_11_	*C*_22_	*C*_33_	*C*_44_	*C*_55_	*C*_66_	*C*_12_	*C*_13_	*C*_23_
0	37.57	79.18	26.45	9.74	32.58	13.70	6.32	11.52	30.55
5	98.55	110.56	54.78	19.39	59.38	29.01	13.72	24.31	49.38
10	139.94	133.80	81.58	26.95	83.87	40.56	19.49	28.63	63.20
15	183.45	148.02	106.82	34.25	107.81	50.32	31.26	39.41	71.95
20	219.94	165.14	131.28	41.0	130.86	58.65	43.14	46.95	83.17
25	249.10	181.29	163.68	43.63	153.85	66.62	52.39	54.58	100.40
30	268.65	197.04	183.77	44.50	175.11	73.99	58.71	56.61	116.15
33	295.59	208.98	215.13	48.26	189.81	79.53	60.49	60.56	132.99
34	290.94	210.78	217.15	51.52	191.48	82.20	61.79	63.91	144.75
35	295.94	214.30	219.96	52.50	194.33	83.83	63.34	65.00	147.35
40	326.33	232.96	234.67	57.43	207.96	91.55	74.71	74.71	160.73
45	351.55	254.03	252.23	61.27	220.23	98.09	78.08	83.28	176.74
50	375.67	273.37	264.88	65.51	233.31	103.39	83.64	88.42	190.89
